# 

**Published:** 1896-06-13

**Authors:** 


### EARLY EDITION.

**Notices of Births, Deaths, and Marriages** are inserted in this column at a charge of 2s. 6d. for an announcement not exceeding four lines.

**Notice to Advertisers and Subscribers.**
All Advertisements, Orders for Copies of THE HOSPITAL and Business Communications should be addressed to **THE MANAGER, (Not to The Editor),** THE HOSPITAL, 428, Strand, London, W.C.
The Cost of Subscription, post free, is as follows:—Per week, 2½d.; per quarter, 2s. 8d.; per half-year, 5s. 3d.; per annum, 10s. 6d. Foreign:—Per Annum, 15s. 2d.; payable, in all cases, in advance.

NOW READY.
**"Burdett's Hospitals and Charities, 1896."**
Seventh year of publication. Over 1,000 pp. Scarlet cloth, gilt lettered, Price 5s. A most complete guide to British, Colonial, Indian, and American Hospitals, Dispensaries, Nursing and Convalescent Institutions, and Asylums. A few complete sets of the preceding six issues of the "Annual" may still be obtained on application to the Publishers, THE SCIENTIFIC PRESS (Limited), 428, Strand, London, W.C.

### NOTICE TO CORRESPONDENTS.

All MS., Letters, Books for Review, and other matters intended for the Editor should be addressed THE EDITOR, THE LODGE, PORCHESTER SQUARE, LONDON, W.

The Editor cannot undertake to return rejected MS., even when accompanied by stamped directed envelope.

**NOTICE.—Vol. XIX. of "The Hospital" (October, 1895, to March, 1896), in handsome cloth binding, is now ready and on sale at the Office of this Journal. Price 7s. 6d. Cases for binding the Numbers contained in this Volume, 1s. 6d. Index to Vol. XIX., 2½d., post free.**
**The Monthly Part for June is now ready, price 6d., by post, 8d.**

### THE STUDENT OF MEDICINE.

### APPOINTMENTS.

P. Lambert, M.R.C.S., L.R.C.P., appointed Medical Officer for the No. 3 District of the Newbury Union. R. Lyddon, M.R.C.S.Eng., L.S.A Lond., appointed Medical Officer of Health to the Urban and Port Sanitary Authorities, Deal. W. R. Meyer, L.S.A., appointed Medical Officer for the No. 4 (Hurstpierpoint) District of the Cuckfield Union. A. T. Morrison, M.B., R.U.I., B.Ch., appointed District Medical Officer of the Aylesbury Union.

### VACANCIES.

The following vacancies are announced this week, the amount of salary in each case being given after the name of the institution :—

### Resident Medical Officers.

Brentford Union.—Medical Superintendent of Infirmary and Medical Officer of Workhouse and Schools. Salary £250 per annum, with furnished residence in the infirmary, rations, washing, &c., or £300 with furnished residence, washing, &c., but without rations. Must be not less than 28 nor more than 40 years of age, and doubly qualified. Also Assistant Medical Officer. Salary £100 per annum, with furnished apartments in the infirmary, rations, washing, &c.; unmarried; doubly qualified. Applications on forms provided to be sent to William Stephens, Clerk to the Guardians, Union Office, Isleworth, W., by June 16th.

Chester General Infirmary.—Visiting Surgeon; must be doubly qualified. Appointment for not more than two years. Salary to commence at £90 per annum, with board and residence. Applications to the Chairman of the Board of Management, Secretar 's Office, 29, Eastgate Row, North Chester, by June 27th.

Hereford County and and Cit y Lunatic Asylum.—Assistant Medical Officer. Salary £100 per a nnum, with board, lodging, and washing. Applications to Dr. Morri son, Medical Superintendent, by June 13th.

Lewes Dispensary and Infi rmary, and Victoria Hospital, Lewes.—Resident Medical Offi cer; doubly qualified. Salary, £110 per annum, with furnishe d apartments, coal, gas, and attendance. Applications to R. Blaker, Honorary Secretary, by June 26th.

London Temperance Hospital, Hampste ad Road, N.W.—Assistant Resident Medical Officer; must possess registrable qualifications in Medicine and Surgery. Appointment for six months. No salary, but residence, board, and washing. Applications to A. W. Bodger, Secretary, by June 17th.

Noble's Isle of Man General Hospital and Dispensary, Douglas, Isle of Man.—Resident House Surgeon; must be single, and doubly qualified. Salary, £90 per annum, with apartments, gas, coals, and washing free. The House Surgeon is usually appointed by the Committee of the House of Industry as Medical attendant to that inst tution at a salary of £10 a year. Applications to F. B. Fleming, Honorary Secretary, by June 16th.

Parish of Dundee Combination.—Resident Medical Officer for the Dundee Combination Poorhouses. Salary £80 per annum, with board and furnished apartments. Applications to James Kyd, Clerk of Parish Council, by June 13th.

Royal Free Hospital, Gray's Inn Road, W.C.—House surgeon; doubly qualified. Appointment for six months, but the holder is eligible for re-election for a further period of six mor ths. No salary, but board, residence, and washing provided. Applications to C. W. Thies, Secretary, by June 27th.

### Non-Resident Medical Officers.

Bedford College (for Women), 8 and 9, York Place, Baker Street, W.—Professorship in Hygiene. Applications to the Honorary Secretary by June 16th.

Hartismere Union.—Medical Officer and Public Vaccinator for the Mendlesham District. Salary will be at the rate of £60 per annum, exclusive of vaccination fees, and such extra fees as are allowed under the consolidated orders of the Local Government Board, the average of which for the last three years is £40 per annum. Applications to John Bond, Clerk, Board Room, Eye, Suffolk, by June 12th.

Hospital for Sick Children, Great Ormond Street, W.C.—House Surgeon to Out-patients, non-resident. Appointment for six months, but eligible for re-election for a second term of office. Salary 25 guineas. Applications to the Secretary by June 16th.

Parish of Creich, Sutherland.—Medical Officer and Vaccinator. Salary as Medical Officer, £45 per annum; as Vaccinator, statutory fees and allowance of £5 in addition. Applications to David Ross, Inspector of Poor, Bonar Bridge, Sutherland, by June 18th.

Parish of Glenelg.—Medical Officer for the Northern Division of Parish. Salary £110 per annum with free house and garden. Gaelic a recommendation. Applications to D. McLure, Inspector of Poor, by June 30th.

Parish of Kincardine, Ross.—Medical Officer and Vaccinator. Salary as Medical Officer, £42 10s. per annum; as Vaccinator, the statutory fees with an allowance of £5 in addition. Applications to Mr. G. G. Macleod, Chairman of the Parish Council, by June 19th.

St. George's and St. James's Dispensary, 60, King Street, Regent Street, W.—Surgeon; must be M. or F.R.C.S Eng. Applications to St. Leger Bunnett, Secretary, by June 20th.

